# Maxillary and Mandibular First Premolars Showing Three-Cusp Pattern: An Unusual Presentation

**DOI:** 10.1155/2013/734143

**Published:** 2013-02-12

**Authors:** Ramakant Nayak, Vijayalakshmi Kotrashetti, Aarati Nayak, Viraj Patil, Mayuri Kulkarni, Pradeep Somannavar, Jagadish Hosmani

**Affiliations:** ^1^Department of Oral Pathology and Microbiology, Maratha Mandal's NGH Institute of Dental Sciences and Research Centre, Belgaum, Karnataka 590010, India; ^2^Department of Periodontology, Maratha Mandal's NGH Institute of Dental Sciences and Research Centre, Belgaum, Karnataka 590010, India; ^3^Department of Prosthodontics, Maratha Mandal's NGH Institute of Dental Sciences and Research Centre, Belgaum, Karnataka 590010, India

## Abstract

Dental anatomy is the study of morphology of various teeth in human dentitions. The application of dental anatomy in clinical practice is important, and dentist should have a thorough knowledge regarding the morphology of the teeth. At times as a result of genetic variation, environmental factors, diet of an individual and race, variations in the morphology of the teeth can be observed. These variations have been extensively studied by the researcher in the field of anthropology to define a particular race. The most commonly observed changes include peg-shaped laterals, shovel-shaped incisors, and extra cusp on molar. Common variations documented with regard to maxillary and mandibular first premolars are the variation in the number of roots. But the variations with respect to crown morphology are few. We report a first documented unusual presentation of maxillary and mandibular first premolars with three-cusps pattern in a female patient.

## 1. Introduction 

Dental anthropology is the study of the origin and variations in the human dentitions. These structural variations are used to determine a population or a race. Dental anthropologic structures useful in identification include metric and nonmetric traits. The study of dental morphology (nonmetric trait) is easily observed and documented. Nonmetric dental traits (NDTs) are of value because they possess high taxonomic value and have been used to estimate biological relationships among diverse population which allows comparative analysis of the historical, cultural, biological development of primitive and modern human groups. NDT can be used to evaluate population differences according to microevolutionary processes which in turn give information about racial variations among the population. Thus it is important to describe systematically the morphological variation in each person's clinical dental history. The most commonly studied traits include study of cusp size, number and location, occlusal pattern, root configuration, and number of roots [1, 2].

Maxillary first premolars are described morphologically as showing two cusps and two roots, whereas the mandibular first premolars shows presence of two cusps and one root, with lingual cusp being rudimentary in most cases. Superimposed on these basic shapes of teeth are minor morphological variations that affect both deciduous and permanent teeth [3, 4]. Such variations are inherited and dependent on many genes, culture, conditions of life, diet, and adaptation processes [4]. The common variation documented in the morphology of maxillary first premolar is presence of three root canals with the incidence of 5-6% [5]. Donald HM found morphological variations in maxillary first premolar in two girls and one boy of Papago Indians. He observed that buccolingual dimension of the crown was increased when compared to second maxillary premolar. The buccal cusp was affected in the study population, which showed presence of hypertrophied medial occlusal paracone ridge. Similarly Brabant et al. noted increased buccolingual dimension of maxillary first premolars due to the presence of supernumerary cusp on the buccal surface of the paracone [6]. Apart from these reports which were documented in late 1960s, no other cases have been documented reporting variation in the crown morphology of maxillary first premolar. Presence of three-cusp pattern in mandibular second premolar is a normal phenomenon [3, 4]. Common variation in the crown morphology observed in this tooth is presence of four cusps [4] and tubercle [1]. The reported variation in the crown morphology of mandibular first premolar varies from no lingual cusp to as many as four lingual cusplets [7].

In this paper we report first unusual case showing three cusp crown pattern in the maxillary and mandibular first premolars in a female patient.

## 2. Case Report

A 46-year-old female patient reported with a chief complaint of draining sinus in the alveolar mucosa of 16. On examination, 16 showed presence of metal crown restoration which was root canal treated about 5 years back. Silver amalgam filling restoration with 17 and 37 was present. Porcelain fused to metal bridges was present with 45, 46, 47 and 24, 25, 26, 27 and porcelain fused to metal crown on 34.

**Figure 1:**
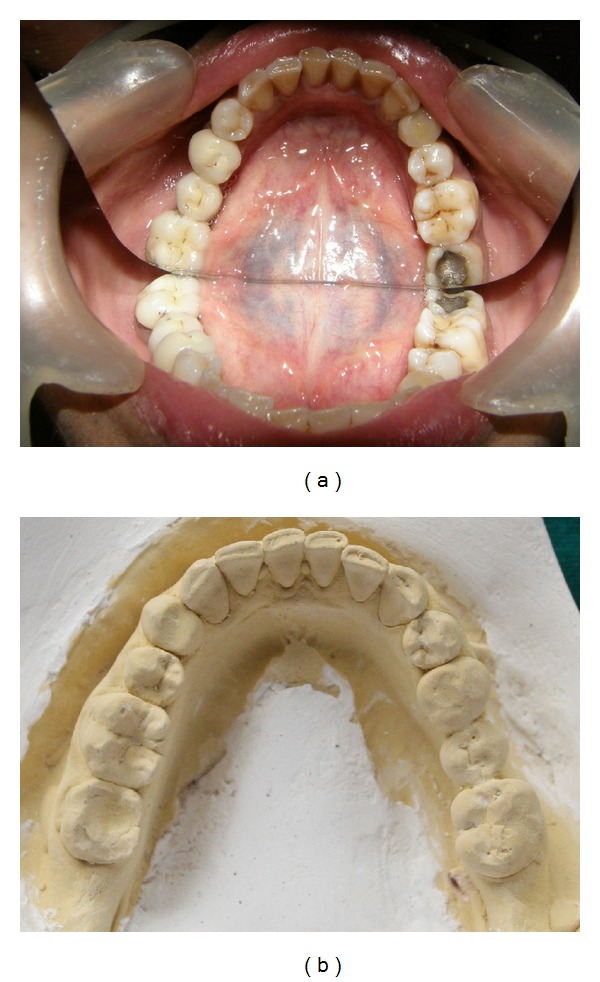
(a) Mirror image photograph showing 44 with mesiolingual and distolingual cusp. (b) Photograph of mandibular cast showing 44 with typical “Y” shaped occlusal groove with 3 cusps resembling mandibular second premolar.

**Figure 2:**
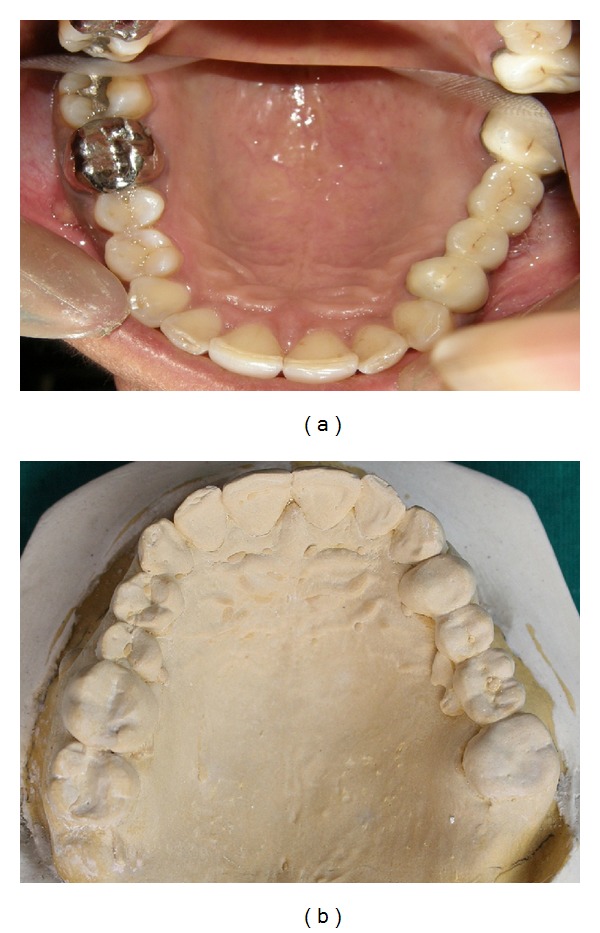
(a) Mirror image photograph showing 14 with mesiolingual and distolingual cusp. (b) Photograph of maxillary cast showing 14 with typical “Y” shaped occlusal groove, 3 cusps and a prominent lingual groove.

**Figure 3:**
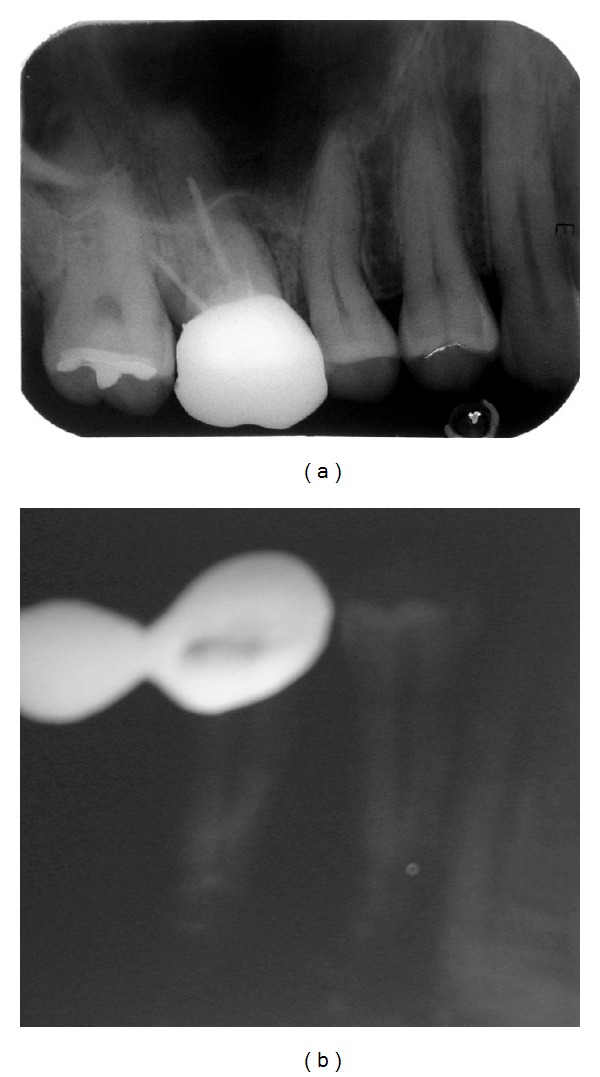
(a) Photograph of intraoral periapical radiograph of 14 showing normal root morphology. (b) Photograph of intraoral periapical radiograph of 44 showing normal root morphology.

We also noticed an unusual variation in the crown morphology of 14 (right maxillary first premolar) and 44 (right mandibular first premolar). Both the crowns showed three-cusp pattern showing “Y” shaped occlusal groove. There was presence of one buccal cusp and two lingual cusps, that is, mesiolingual and distolingual separated by the groove which was extending on to the lingual surface and appeared as lingual developmental groove. On the contralateral side, we examined whether the same phenomenon exists but 34 was root canal treated with porcelain fused metal crown and 24 had crown which was an abutment for bridge. In case of mandibular first premolar the possibility of transposition could not be ascertained as 45 was an abutment for a bridge. Of the two lingual cusps in 44, the mesiolingual cusp was more prominent (Figures 1(a) and 1(b)) and in case of 14, the mesiolingual cusp was marginally larger than the distolingual cusp (Figures 2(a) and 2(b)). Occlusal aspect of 14 and 44 showed well developed marginal ridges and prominent mesial and distal fossa. The mesial and distal occlusal developmental groove was prominent and was extended till the marginal ridge in 14, whereas it was less prominent in 44. Occlusion was class I, and there was no interference in the occlusion with opposing teeth with respect to 14 and 44. The buccal cusp of 44 occluded in the central pit of 14 (at the junction of occlusal and palatal groove). The distolingual cusps of both the teeth were not in contact with the opposing teeth, as they were small in size. The emerging pattern and timing of teeth were normal. The intraoral periapical radiograph of 14 did not show any root variations (Figures 3(a) and 3(b)). The thickness of enamel and dentin appeared to be normal. The crown size of the teeth was measured using digital vernier caliper on dental cast. The mesiodistal dimension of 14 was 6.62 mm and buccolingual was 7.84 mm. The mesiodistal dimension of 44 was 5.85 mm and buccolingual was 7.86 mm.

## 3. Discussion 

The commonest morphological variations described in dental anatomy include presence of shovel-shaped maxillary central incisor, peg-shaped maxillary lateral incisor, accessory cusp on the maxillary first permanent molar, additional cusp on the mandibular second premolar giving a total of four cusps to the tooth, reduced size or absence of a distopalatal cusp on the maxillary second molar [4]. “At times certain of these changes go unnoticed and will not be documented in routine dental practice.”

Knowing some common variations occurring in tooth morphology about each individual tooth can help in performing dental treatment and also can be used for anthropological research for identification of population [2].

We report a first ever documented case of maxillary first premolar and mandibular first premolar showing three cusps. It is one of the rare variations observed. Both the teeth showed classic presence of mesiolingual and distolingual cusp. Probably the same morphology of crown was present on the contralateral side but we could not confirm as patient had porcelain fused to metal crowns on 34 and 24 was an abutment for bridge. The size of the teeth appeared larger mesiodistally when compared to normal average dimension [3]. 

The etiology for extra cusp formation is unknown. However, earlier it was thought to be due to over-activity of the dental lamina. But now it is believed that PAX and MSX genes are responsible for variation in shape of the teeth [8]. Extra cusps develop due to abnormal proliferation and folding of a portion of inner enamel epithelium (IEE) along with adjacent ectomesenchymal cells of the dental papilla into the stellate reticulum of the enamel organ during bell stage of tooth formation.The resultant formation is defined as either tubercle or supplemental solid elevation on some portion of the crown surface [9].

Current embryological evidence suggests that primary and secondary enamel knots direct the folding of IEE which determines the characteristic morphology of the crown. Enamel knots begin to form in the cap stage of tooth development and location of primary enamel knot coincides with the presumptive apex of the first-forming cusp, and subsequently secondary enamel knots develop during the bell stage, which coincides with the number and position of the other presumptive cusps [10].

Enamel knots are transitory condensations of IEE situated in the stellate reticulum atop the IEE projecting toward dental papilla. Enamel knot acts as a signaling center and consists of nondividing cells, and it stimulates rapid proliferation of adjacent dental epithelium though they themselves are nonproliferative. This relationship seems to be central to the formation of the tooth cusps [10, 11]. Localized differences in cell proliferation cause folding of the IEE, where the enamel knots define the number and position of the presumptive cusps tips. During this process, topographic difference in proliferation rates of the epithelium accounts for the angularity of the cusps and also for differences in cusp height. This indicates that activator from the primary enamel knot regulates the expression of secondary enamel knots. The resultant cusp morphogenesis and positions appear to be determined sequentially, and cusps that form late in development, after the main cusps, are typically small.The secondary enamel knots disperse after formation of the cusp tips, which indicates termination of crown morphogenesis. Furthermore, the actual number of cusps realized in each tooth is also determined by the initiation of root formation [10, 12]. This may be the probable reason for the presence of three cusps in our case.

It is also stated that maxillary premolars and mandibular first premolar develop from four lobes (mesial, distal, buccal, and palatal), whereas mandibular second premolar which often has two lingual cusps develop from five lobes (mesial, buccal, distal, mesiolingual, and distolingual lobes) [3]. Probably in the present case the maxillary first premolar and mandibular first premolar have developed from five lobes thus showing three cusps.

## 4. Conclusion

We present a first reported case of three-cusp pattern of maxillary and mandibular first premolar. This can be considered as one of the morphological variations which can be seen and is not suggestive of any kind of a developmental anomaly. They are normal morphological features of the dentition. As a dentist we should be aware of such morphological variations observed during routine dental examination and one should not be very dogmatic about the standard morphological features of the teeth. Proper documentation of these variations may help anthropologists in their study of a population.
